# Challenges in studying statewide pedestrian injuries and drug involvement

**DOI:** 10.1186/s40621-018-0173-8

**Published:** 2018-12-03

**Authors:** Elizabeth D. Nesoff, Charles C. Branas, Silvia S. Martins

**Affiliations:** Department of Epidemiology, Columbia University Mailman School of Public Health, 722 W168th St, 5th floor, New York, NY 10032 USA

**Keywords:** Surveillance, Pedestrian injury, Drug use, Marijuana, Alcohol

## Abstract

**Background:**

Increasing U.S. rates of pedestrian injuries could be attributable in part to changing policies and attitudes towards drugs and associated increases in use, yet drug use has not been investigated widely as a risk factor for pedestrian injury. This study details challenges to investigating drug-involved pedestrian crashes using existing surveillance systems.

**Methods:**

Using California police reports from 2004 to 2016, we performed simple linear regression with the proportion of data that was missing by year for drug and alcohol use as the outcome of interest. We also explored differences in the relative proportion of missing data across sex, race, and age groups through simple logistic regression. Finally, we compared missing data for alcohol and drug use indicators for pedestrians and drivers.

**Results:**

From 2004 to 2016, 182,278 pedestrians were involved in crashes across California. Only 1.22% (*n* = 2219) of records indicated drug use, and 98% had missing data for drug use; the proportion of missing data did not change over time (b = − 0.040, *p* = 0.145, 95% CI = (− 0.095, 0.016)). The proportion of missing values for alcohol use increased each year (b = 0.49, 95% CI = (0.26, 0.72), *p* = 0.001). Driver drug and alcohol use indictors showed similar data missingness, and missing data did not show significant variation over time. Hispanics were more likely to have missing data for drug use compared to Whites (OR = 0.61, *p* < 0.001, 95% CI = (0.56, 0.67)), and Blacks were more likely to have missing data for alcohol use compared to Whites (OR = 0.87, *p* < 0.0001, 95% CI = (0.84, 0.91)).

**Conclusions:**

Drug use may be a key contributing factor to pedestrian injury, but drug use remains consistently and largely unmeasured in existing surveillance systems. Without better collection of drug and alcohol data, monitoring trends in drug-involved pedestrian injury will not be feasible.

## Introduction

The changing landscape of drug use in the United States has potentially significant consequences to health and safety. Expansion of legal marijuana use has been associated with increased motor vehicle crashes, (Li et al. 2012; Salomonsen-Sautel et al. 2014) and the national prevalence of marijuana positivity in fatally-injured drivers reportedly tripled from 1999 to 2010 (Brady and Li 2014). Narcotic, stimulant, and depressant use has also been shown to more than double drivers’ fatality risk (Li et al. 2013). At the same time, pedestrian fatality increased by 25% from 2010 to 2015 (National Center for Statistics and Analysis 2017), yet drug use has not been nearly as widely investigated as alcohol as a risk factor for pedestrian injury.

For example, marijuana intoxication impacts the cognitive functioning necessary for safe street crossing and judgement of traffic patterns while on foot. Marijuana use affects basic motor coordination to more complex tasks such as the ability to plan, solve problems, and make decisions (Crean et al. 2011). Implementation of medical marijuana laws has been associated with increased probability of daily marijuana use among adults age 21 and over and decreased perception of marijuana as harmful or risky (Wen et al. 2015; Pacula et al. 2015; Carliner et al. 2017). Consequently, the risk to pedestrians attributable to marijuana use could be significant. To date, there has been no research conducted in the United States on injury risk to pedestrians who use marijuana or drugs other than alcohol.

This paper investigates the challenges to study of drug-involved pedestrian crashes using existing surveillance systems. Previous research indicates national surveillance systems which monitor fatal motor vehicle crashes may not effectively capture drug use (Romano et al. 2017), but data sources which monitor non-fatal crashes have not been evaluated. Furthermore, discussion of drug use as a risk factor for non-fatal injury has been lacking. Most of this research has looked at fatal crashes only (and intoxication among drivers primarily), which are relatively rare and make up a small proportion of pedestrian injuries (National Center for Statistics and Analysis 2017). Inquiry into drug-involved pedestrian injury is hampered by technological limitations in field sobriety testing (Wong et al. 2014; Walsh et al. 2004; Karschner et al. 2009). Variations in individual tolerance and the pharmacological characteristics of different drugs present further difficulties (Li et al. 2013; Walsh et al. 2004). For example, an inactive metabolite of THC in blood and urine can be detected for weeks after marijuana use (Ramaekers et al. 2004). For these reasons, pedestrian drug intoxication status may be misidentified in existing surveillance systems.

Our study investigates changes in the proportion of missing data for drug and alcohol use indicators in California over time. As California has been on the forefront of the changing landscape of marijuana policy—enacting legislation decriminalizing marijuana for medical use in 1996 (State of California 1996) and recreational use in 2016 (State of California 2016)—it is an ideal location to examine the impact of policy changes and associated drug use on pedestrian injury. We chose alcohol use surveillance as a comparison measure as alcohol is an established drug use risk factor for pedestrian injury, (Oxley et al. 2006; Dultz et al. 2011) and preventing alcohol-intoxicated pedestrian injury has been the focus of multiple health initiatives (Lenné et al. 2007; Hutchinson et al. 2010; Corben et al. 1996). Because of increasing awareness and accessibility of marijuana, increasing awareness of opioid addiction, and potentially-associated increases in pedestrian-involved motor vehicle crashes, we investigated missing data in drug and alcohol use indicators and included race, gender, and age comparative breakdowns. We also investigated differences in missing data trends for drug and alcohol use indicators between pedestrians and drivers who hit pedestrians.

## Methods

### Data source and measurement

We examined California police collision reports from 2004—the year that medical marijuana laws were expanded to allow for marijuana collectives, also known as dispensaries (California Health and Safety Code n.d.)—to 2016 extracted from Statewide Integrated Traffic Reporting System (SWITRS) (California Highway Patrol n.d.). SWITRS, maintained by the California State Highway Patrol (CHP), provides a rich data source for exploring pedestrian injury over time. California law requires all police to submit a report to CHP for every traffic collision that results in an injury or death (California Highway Patrol 2017). SWITRS provides demographics on all parties involved, crash details, and GPS coordinates of crash locations. SWITRS excludes intentional homicides, suicides, and off-road incidents on private property (California Highway Patrol 2017). As this study analyzes data from a de-identified and publicly-available data source, it is considered exempt from human subjects research.

The CHP 555 Traffic Collision Coding Form, filled out for every crash resulting in an injury, allows for investigation into drug and alcohol use at the time of a crash. Alcohol and drug use are captured under “Sobriety–Drug Physical.” Options include “had not been drinking; had been drinking, under influence; had been drinking, not under influence; had been drinking, impairment unknown; under drug influence; impairment–physical; impairment not known; not applicable; sleepy/fatigued.” Investigators are instructed to mark only 1 or 2 items. CHP’s Collision Investigation Manual defines “had been drinking” and “under drug influence” broadly. For example, “under drug influence” is defined as, “The involved party appears to be under the influence of a drug other than alcohol” (California Highway Patrol 2017). “Not applicable” indicates a motor vehicle that was parked, driverless, or otherwise unoccupied at the time of the crash and should not be marked for an involved party (California Highway Patrol 2017).

However, the Collision Investigation Manual does not provide detail for investigating pedestrian intoxication, and there is no discussion of how drug use should be tested or what drugs were used at the time of the crash. Officers are instructed to “mark the appropriate box in the pedestrian’s Party column as it relates to the pedestrian’s sobriety/drug/physical impairment status” (California Highway Patrol 2017). If more than one pedestrian is involved in a crash, only the actions of the first pedestrian injured or otherwise involved just prior to the collision should be recorded; it is unclear if the same goes for other pedestrian characteristics, including intoxication. There is a separate section for a narrative summary describing the intoxication investigation, but these are not included in SWITRS. First, details on the party’s actions—including “objective symptoms of intoxication; the odor of alcoholic beverage and the state of their eyes, speech, hand-eye coordination, balance, etc.”—which prompted the investigation should be included in the narrative, as well as “how the party was determined to be the driver” (California Highway Patrol 2017). Then, the officer should indicate whether a Field Sobriety Test was administered and whether the party was arrested. Finally, “if determined to be not under the influence, state how the alcohol and/or drug consumption was established and the method used to determine the party was not under the influence” (California Highway Patrol 2017). No instructions for assessing drug intoxication or type of drug other than alcohol are offered. Data provide no detail on type of drug used or if the drug was illicit or prescription.

Although the Traffic Collision Coding Form lists drug and alcohol intoxication as one measure, SWITRS presents drug and alcohol measures as discrete variables. Alcohol use is measured as “had not been drinking; had been drinking, under influence; had been drinking, not under influence; had been drinking, impairment unknown; impairment unknown; not applicable.” Drug use is combined with other physical impairments, and category choices included “under drug influence; impairment–physical; impairment unknown; not applicable; sleepy/fatigued.” There is no “had not been using drugs” option for drug use.

### Data analyses

Analyses were carried out using STATA 13. To look at trends in missing data over time, we created a new variable for drug use recoded into three categories: “Under drug influence” was unchanged, while physical impairment and sleepy/fatigued were collapsed into “other physical impairment.” “Impairment unknown,” “not applicable,” and blank items were coded as “missing.” Alcohol use was recoded by collapsing the three “had been drinking” categories into one item, while “had not been drinking” was unchanged. “Impairment unknown,” “not applicable,” and blank values were recoded as “missing.”

Race/ethnic groups were reported in SWITRS as “White, Black, Hispanic, Asian, Other” (California Highway Patrol 2017). The Collision Investigation Manual instructs officers to “use observation and their best judgment only to determine the party’s race;” (California Highway Patrol 2017) consequently, race/ethnic groups are subjective and not based on self-report. Sex was likewise reported as male, female, or “not stated.” Age was recorded as a continuous variable from 0 to 105 years. We recoded age into three groups to examine missing data among age groups most at risk for pedestrian injury (National Center for Statistics and Analysis 2017; Nesoff et al. 2018): Children age 0 to 18 years and older adults age 65 and older, with adults age 19 to 64 as reference. For the drivers comparison group, we excluded drivers who had left the scene of the crash, coded as “hit and run” drivers by SWITRS, because no information was available on their drug and alcohol use at the time of the crash.

To examine trends in missing data over time, we first explored the distribution of all variables of interest. We then performed simple linear regression with the proportion of data that was missing by year as the outcome of interest. We also tested for different spline terms to best model the trend in missing data over time. We examined differences in the relative proportion of missing data across sex, race, and age groups by performing logistic regression with a binary response variable of 0 indicating the response was left blank and 1 indicating any explicit response (e.g., “had been drinking” and “had not been drinking” were both considered a response and coded as 1, while “not applicable” and “impairment unknown” were considered non-responsive and coded as 0).

## Results

From 2004 to 2016, 182,278 pedestrians were involved in crashes across California; on average, there were 14,021.4 injuries per year (sd = 572.3). The overall trend in total injuries was not significant (b = − 10.19, *p* = 0.822, 95% CI = (− 107.47, 87.09)). However, there was a significant decrease in pedestrian injuries prior to 2011 (b = − 203.63, *p* = 0.002, 95% CI = ((− 310.07, − 97.18)), and a significant increase after 2011 (b = 299.32, p = 0.002, 95% CI = (144.54, 454.09)). The mean age of injured pedestrians across all years was 43.4 years (sd = 88.5). A majority of injured pedestrians were coded as “Hispanic” (38.2%, *n* = 69,654) and male (56.5%, *n* = 102,949). Race/ethnicity category for pedestrians was missing for 6.7% (*n* = 12,231) of responses, and sex category was missing for 0.8% (*n* = 1423) of responses.

**Fig. 1:**
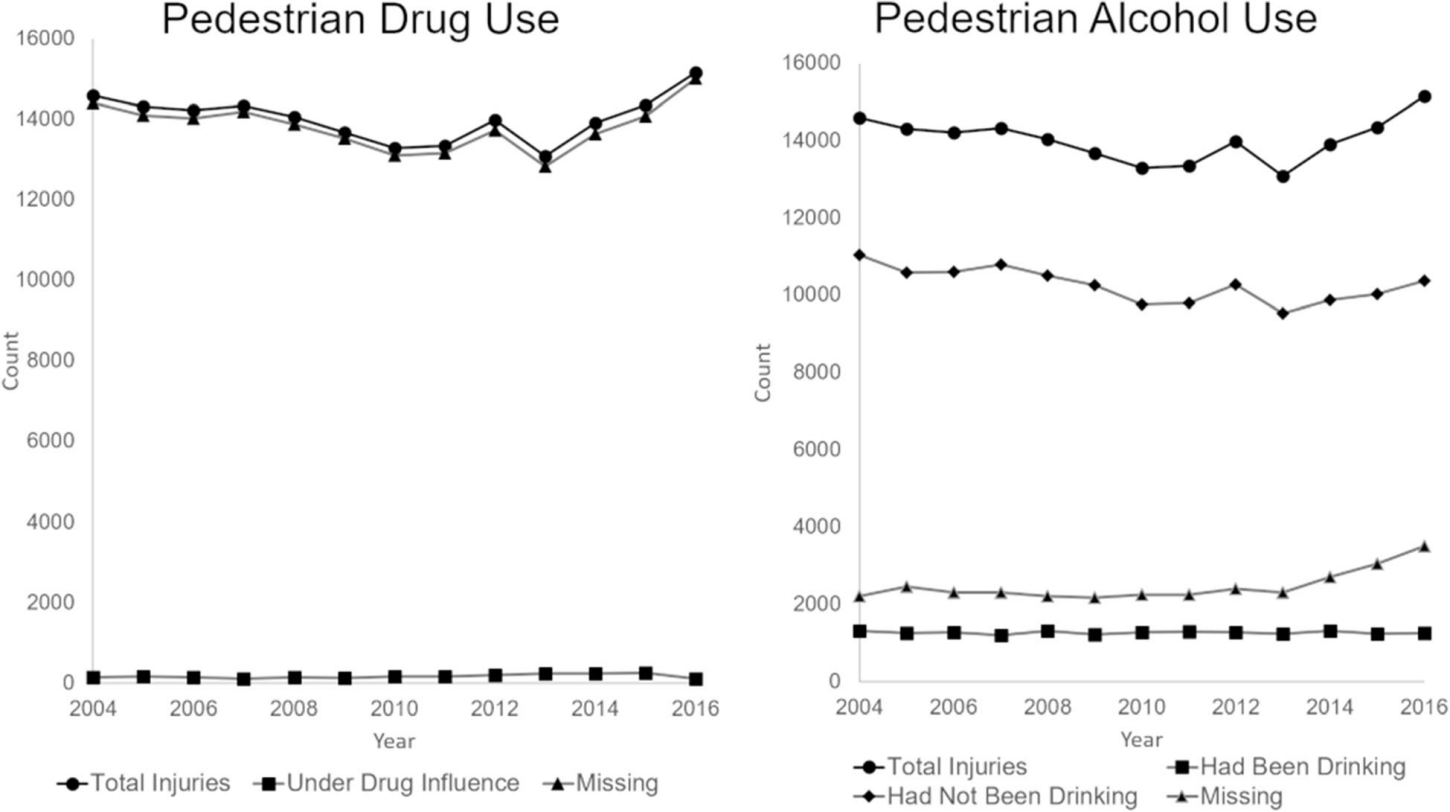
Description of drug and alcohol use indicators for injured pedestrians, 2004–2016

Only 1.22% (*n* = 2219) of cases across all years indicated drug use (Fig. 1). Almost 90% (*n* = 159,697) of total records were left blank, 6.5% (*n* = 11,855) listed drug impairment as unknown, and 4.5% (*n* = 8112) were listed as “not applicable.” Alcohol consumption was indicated for 9.1% (*n* = 16,544) of injured pedestrians across all years, while 73.2% (*n* = 133,476) were listed as “had not been drinking.” For alcohol involvement, 6.8% (*n* = 12,340) of total records were left blank, 6.5% (*n* = 11,842) were listed as “impairment unknown,” and 4.4% (*n* = 8075) were listed as “not applicable”—resulting in missing indicators for 17.7% of pedestrians across all years combined.

**Fig. 2:**
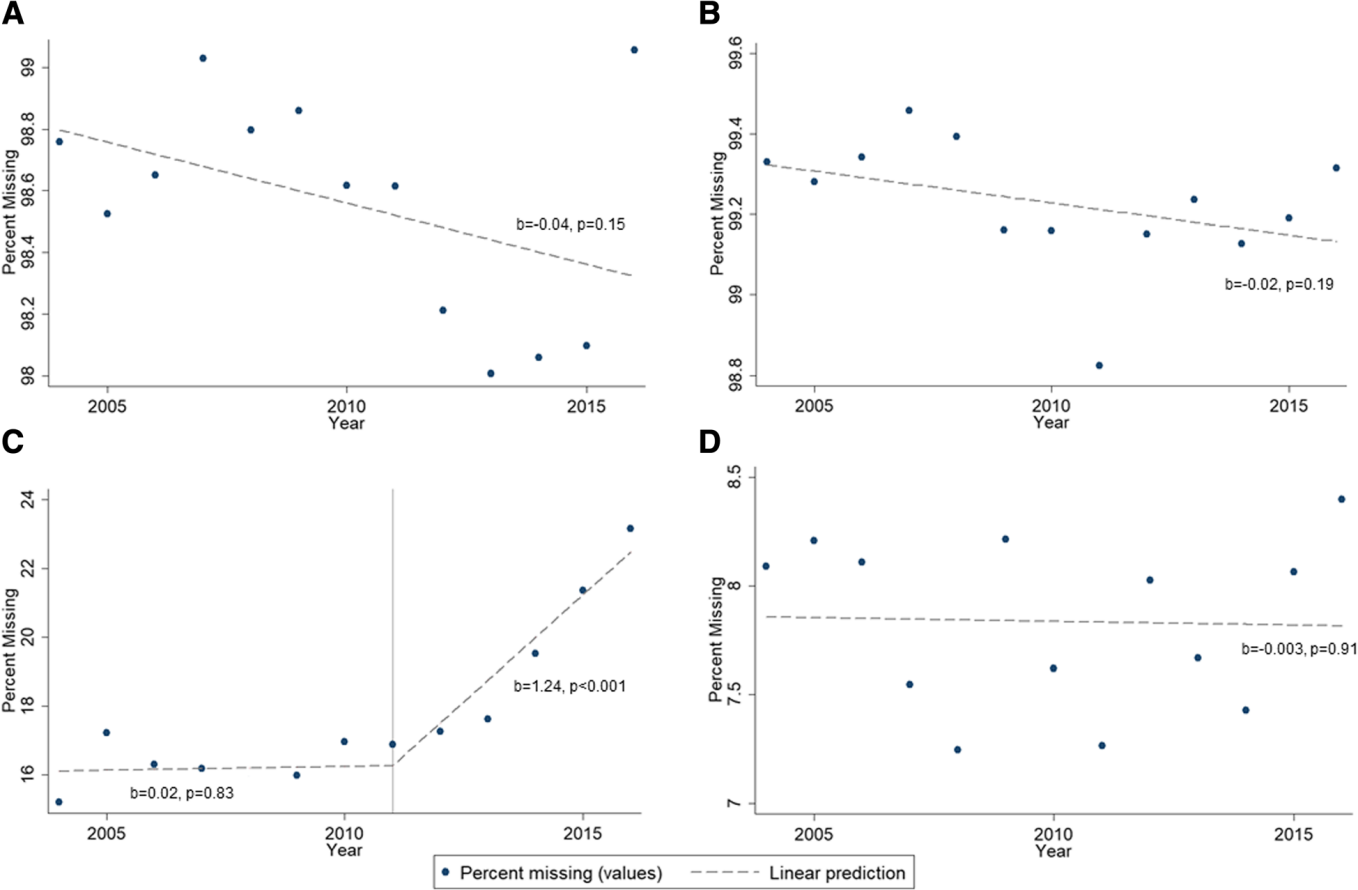
Trends in missing data for drug and alcohol use indicators for pedestrians and drivers, 2004–2016. **a** Pedestrian drug use. **b** Driver drug use. **c** Pedestrian alcohol use. **d** Driver alcohol use

The percent of missing data for drug use showed no significant changes over time (b = − 0.040, *p* = 0.145, 95% CI = (− 0.095, 0.016)) (Fig. 2). The percent of pedestrians listed as using drugs showed a positive trend from 2004 to 2016, but this change was not significant (b = 0.043, *p* = 0.111, 95% CI = (− 0.012, 0.097)). Missing data for alcohol use significantly increased over time. The overall trend from 2004 to 2016 showed a 0.49% increase in missing data each year (95% CI = (0.26, 0.72), *p* = 0.001). However, the missing data increase was concentrated after 2011. Prior to 2011, the yearly increase in missing data was not significant (b = 0.02, *p* = 0.83, 95% CI = (− 0.20, 0.24)). After 2011, the percent of missing data increased by 1.24% each year (*p* < 0.001, 95% CI = (0.92, 1.56)). The proportion of pedestrians listed as “had not been drinking” showed significant decreases over time (b = − 0.49, *p* < 0.001, 95% CI = (− 0.67, − 0.32)). This variable also showed a changing trend after 2011. Prior to 2011, the decrease in pedestrians listed as “had not been drinking” was not significant (b = − 0.15, *p* = 0.146, 95% CI = (− 0.35, 0.06)). After 2011, the proportion of pedestrians listed as had not been drinking decreased by 1.05% each year (p < 0.001, 95% CI = (− 1.35, − 0.75)). The proportion of pedestrians listed as “had been drinking” did not show significant changes over time (b = 0.004, *p* = 0.918, 95% CI = (− 0.07, 0.08)).

There were significant discrepancies in missing data by race, gender, and age groups for both drugs and alcohol (Table 1). The odds of missing data for drug use were significantly greater for all ethnic groups compared to Whites except for Blacks when controlling for year; there was no significant difference in the odds of missing data between Black and White pedestrians (OR = 0.93, *p* = 0.182, 95% CI = (0.87, 1.04)). For alcohol use, the odds of missing data for Blacks compared to Whites was significantly greater (OR = 0.87, *p* < 0.0001, 95% CI = (0.84, 0.91)) when controlling for year; all other race groups had significantly greater odds of no missing data compared to Whites. For example, the odds of an explicit response for alcohol use for Hispanic pedestrians was 3.7% greater than for White pedestrians (OR = 1.037, *p* = 0.014, 95% CI = (1.007, 1.068)) when controlling for year. Men were significantly less likely to have missing data for drug use (OR = 1.94, *p* < 0.001, 95% CI = (1.78, 2.11)) and significantly more likely to have missing data for alcohol use (OR = 0.90, p < 0.001, 95% CI = (0.87, 0.92)) compared to women, controlling for year. Older (≥65 years) and younger pedestrians (≤18 years) were significantly more likely to have missing data for drug use and significantly less likely to have missing data for alcohol use compared to pedestrians age 19 to 64 years.

**Table 1:** Discrepancies in missing data for drug and alcohol use indicators by selected groups, 2004–2016

	Drug Use			Alcohol Use		
Variable	Odds Ratio^a^	95% CI	*p*	Odds Ratio^a^	95% CI	*p*
Race (ref: White)^b^						
Black	0.926	0.83, 1.04	0.182	0.871	0.84, 0.91	< 0.001
Hispanic	0.608	0.56, 0.67	< 0.001	1.037	1.01, 1.07	0.014
Asian	0.384	0.30, 0.48	< 0.001	1.133	1.07, 1.20	< 0.001
“Other”	0.548	0.48, 0.69	< 0.001	1.080	1.01, 1.15	0.017
Sex (ref: Women)^b^						
Men	1.945	1.78, 2.12	< 0.001	0.896	0.87, 0.92	< 0.001
Age (ref: 19–64)^b^						
0–18 years	0.170	0.15, 0.20	< 0.001	1.164	1.13, 1.20	< 0.001
65+	0.640	0.56, 0.73	< 0.001	1.075	1.03, 1.12	< 0.001

^a^Measures the odds of an explicit response: 1 indicates an explicit response (e.g., “had been drinking” or “had not been drinking”); 0 indicates missing data (e.g., “not applicable,” “impairment unknown,” blank)

^b^Controlling for year

A total of 175,183 drivers were involved in pedestrian crashes from 2004 to 2016, but almost 20% of these (*n* = 34,124) were hit-and-run drivers. Consequently, 141,059 drivers across all years were included. Of these, 99.2% (*n* = 131,695) of driver records were left blank for drug use and only 0.47% (*n* = 831) of drivers were listed as “under drug influence.” In contrast, 88.9% (*n* = 125,364) of drivers were listed as “had not been drinking” and approximately 7.8% (*n* = 11,064) of records were left blank for alcohol use. The percent of missing records for drivers’ drug use (b = − 0.016, *p* = 0.191, 95% CI = (− 0.041, 0.009)) and drivers’ alcohol use (b = − 0.003, *p* = 0.912, 95%CI = (− 0.07, 0.06)) did not show any significant variation over time (Fig. 2). The proportion of drivers listed as “under drug influence” showed a positive trend, but this change was only marginally significant (b = 0.015, *p* = 0.077, 95% CI = (− 0.002, 0.031)). The proportion of drivers listed as “had been drinking” (b = − 0.004, *p* = 0.74, 95% CI = (− 0.027, 0.020)) or “had not been drinking” (b = 0.007, *p* = 0.84, 95% CI = (− 0.07, 0.08)) showed no significant variation over time.

## Discussion

The purpose of this study was to detail challenges to investigating trends in drug-involved pedestrian injury over time. While this study focuses on California’s crash reporting system, the goal was to identify problems with current surveillance systems for drug-involved traffic crashes and not criticize California in particular. Rather, California serves as a model for identifying challenges in monitoring drug-involved crashes from existing surveillance systems. California was chosen as the site of this study because of the evolving landscape of marijuana laws in the state. Therefore, findings from this study are intended to identify challenges in existing injury surveillance systems.

Approximately 90% of records for drug use among pedestrians and 99% among drivers were missing, and trends in missing data did not change over time. The high proportion of missing data for drug use across all years may be related to challenges in detecting marijuana and other drugs in the field (Wong et al. 2014; Walsh et al. 2004). However, roughly 18% of records for alcohol use among pedestrians and 8% for drivers were missing across all years, and the proportion of missing data for pedestrian alcohol use significantly increased over time. National surveillance systems of fatal traffic crashes show similar variability in missing drug use indicators (Romano et al. 2017).

Previous studies of fatal drug-involved motor vehicle crashes using national surveillance systems have limited analyses to jurisdictions with high drug testing rates or used multiple imputation methods to avoid bias related to complete case analysis (Li et al. 2013; Romano et al. 2017). Consequently, there has been little discussion of patterns in missing data for motor vehicle crashes. Missing drug test data in fatal surveillance systems has been attributed to discrepancies across jurisdictions in reporting requirements, quality and frequency of drug testing, method of drug testing, and drugs included in testing (Romano et al. 2017; Berning and Smither 2014). Studies have demonstrated bias in drug and alcohol testing for other forms of intentional and unintentional traumatic injury, but possible bias in drug testing in motor vehicle crash surveillance systems has not been widely discussed (Keyes et al. 2012).

Positive indicators for alcohol use showed a significant decrease over time, and this increase intensified after 2011—meaning that alcohol intoxication was recorded significantly less often over time after 2011. This is concerning as national trends indicate alcohol intoxication has steadily increased among fatally-injured pedestrians since 2010 (National Center for Statistics and Analysis 2017). Furthermore, pedestrians who consume alcohol at the time of a crash experience more severe injuries and suffer longer recovery times compared to sober pedestrians (Dultz et al. 2011; Plurad et al. 2006). Acute measurement of alcohol use at the time of a crash could have immediate consequences for treatment outcomes as intoxication confounds the detection of traumatic brain injury and the anesthetic management of pain (Dultz and Frangos 2013). Consistent surveillance of drug and alcohol use among pedestrians has significant public health consequences for planning effective safety interventions to prevent injury to intoxicated pedestrians.

It is unclear why missing data for alcohol increased after 2011. While there is some argument that the economic downturn starting in 2008 may have contributed as periods of economic decline are associated with decreases in fatal motor vehicle crashes (National Center for Statistics and Analysis 2016), this does not necessarily explain trends in missing data in police reports. There was a significant decrease in total pedestrian injuries prior to 2011, but this was not accompanied by a concurrent decline in missing data. Prior to 2011, rates of missing data for alcohol use among pedestrians were relatively stable. Therefore, the increasing missing data after 2011 cannot be attributed completely to increased vehicle-miles traveled and associated police activity.

All race/ethnic groups showed increased odds of missing data for drug use compared to Whites, and Blacks showed increased missing data for alcohol use indicators compared to Whites. The missing data for Blacks and Hispanics is concerning considering that Blacks and Hispanics disproportionately suffer fatal injury related to excess alcohol consumption for all types of motor vehicle crashes (Keyes et al. 2012), and Blacks and Hispanics experience more pedestrian injuries and greater risk for pedestrian fatality than Whites (Maybury et al. 2010). It is possible that language barriers influenced the higher odds of missing data among certain minority groups as a previous California study found pedestrian injuries to be more frequent in areas where a high proportion of residents did not speak fluent English (Chakravarthy et al. 2010). Further inquiry into the antecedents of discrepancies in surveillance across racial and ethnic groups is necessary and may illuminate strategies for targeted injury prevention programs for groups disproportionately at risk for pedestrian injury and fatality.

Measurement problems related to the CHP protocol for collision investigation and the “Sobriety–Drug Physical” items on the Traffic Collision Coding Form may contribute to missing data for drug and alcohol use. Drug and alcohol use indicators are measured as one item in the coding form but disseminated as discrete variables. SWITRS does not provide a “no drug use” option, potentially increasing the proportion of missing data for the drug use indicator. Furthermore, the Traffic Collision Coding Form instructs officers to fill in only 1 or 2 items for the sobriety measure. Concurrent use of marijuana and alcohol is increasingly common (Yurasek et al. 2017), and concurrent drug and alcohol use has been shown to significantly increase risk for fatal injury among drivers (Li et al. 2013; Chihuri et al. 2017). By limiting officers to only 1 or 2 choices, the protocol may inhibit effective surveillance of co-occurring drug and alcohol use. At the same time, the Collision Investigation Manual does not provide explicit instructions for investigating drug and alcohol use among pedestrians, which may account for the common misuse of the “not applicable” option in these data. Developing an explicit protocol for investigating drug and alcohol use among pedestrians and retraining of law enforcement and paramedics may be necessary to effectively investigate the role of drug use in pedestrian injuries. Adapting the standard field sobriety test to incorporate observable indicators of drug use or new technologies for detecting drug use at the time of a crash may also be viable strategies to improve drug use surveillance (Musshoff et al. 2014; Newmeyer et al. 2017).

## Limitations

This study examines traffic injury surveillance in one state over time. Although efforts have been made on a national level to standardize traffic injury surveillance systems (National Highway Traffic Safety Administration n.d.), data and measurement challenges in California’s injury surveillance system may not be generalizable to other states. A substantial proportion of drivers who hit pedestrians were not included in this study because they fled the scene of the crash before police investigation could take place. It is possible these drivers were more likely to be intoxicated and left the scene as a result. Consequently, missing data for drug and alcohol involvement among drivers may be overestimated. Likewise, pedestrians who were injured but did not summon the police may not be present in these data. Previous studies have shown that Blacks, men, and pedestrians with less severe injuries may be underrepresented in police reports because of the reluctance on the part of some to involve police when they have not been called to the scene of a crash (paramedics in California are not required to report pedestrian injuries to police) (Sciortino et al. 2005). We did not have access to medical reports for the pedestrians included in this study as these records are not publicly available. While it is possible these records would have included more complete drug and alcohol screening results, drug and alcohol screening in trauma care is not routine and may be biased based on patient’s age, sex, or race when conducted at all (Dultz and Frangos 2013; Yuma-Guerrero et al. 2012).

## Conclusions

Drug use may be a key contributing factor to pedestrian-involved crashes, but drug use has remained overwhelmingly unmeasured in existing surveillance systems over multiple consecutive years. Consequently, public health practitioners may be unable to create effective interventions to prevent drug-involved pedestrian injury or make policy recommendations for marijuana legalization or control. Without improving measurement and data collection of drug and alcohol use in current injury surveillance systems, monitoring trends in drug-involved pedestrian injury will not be feasible.

## Abbreviations

CHP: California State Highway Patrol
SWITRS: Statewide Integrated Traffic Reporting System
